# Combined administration of interleukin-2 and 18 with anti-PD-L1 antibody induces CCL5-positive CD8 T cells to suppress liver tumors

**DOI:** 10.1093/pnasnexus/pgag113

**Published:** 2026-04-15

**Authors:** Masamichi Kimura, Kenzaburo Yamaji, Kenichi Harada, Hideya Kawaji, Jun Imamura, Haruki Okamura, Yoshimasa Tanaka, Michinori Kohara, Kiminori Kimura

**Affiliations:** Department of Hepatology, Tokyo Metropolitan Cancer and Infectious Diseases Center Komagome Hospital, Tokyo 113-8677, Japan; Department of Microbiology and Cell Biology, Tokyo Metropolitan Institute of Medical Science, Tokyo 156-8506, Japan; Department of Human Pathology, Kanazawa University Graduate School of Medical Sciences, Kanazawa 920-8640, Japan; Research Center for Genome & Medical Sciences, Tokyo Metropolitan Institute of Medical Science, Tokyo 156-8506, Japan; Department of Hepatology, Tokyo Metropolitan Cancer and Infectious Diseases Center Komagome Hospital, Tokyo 113-8677, Japan; Laboratory of Tumor Immunology and Cell Therapy, Hyogo College of Medicine, Nishinomiya, Hyogo 663-8501, Japan; Center for Medical Innovation, Nagasaki University, Nagasaki 852-8588, Japan; Department of Microbiology and Cell Biology, Tokyo Metropolitan Institute of Medical Science, Tokyo 156-8506, Japan; Department of Hepatology, Tokyo Metropolitan Cancer and Infectious Diseases Center Komagome Hospital, Tokyo 113-8677, Japan

**Keywords:** IL-18, IL-2, CCL5, effector CD8 T cells, hepatocellular carcinoma, immunotherapy

## Abstract

Remarkable progress has been made in cancer immunotherapy, partly because of the development of immune checkpoint inhibitors. However, their efficacy varies across cancer types, with limited response observed in solid tumors, such as hepatocellular carcinoma (HCC). The primary cause of this low efficacy is insufficient infiltration of effector cells, such as cytotoxic CD8^+^ T cells, into the tumor tissue. This study investigated whether recombinant interleukin (rIL)-2, rIL-18, and antiprogrammed cell death-ligand 1 (αPD-L1) antibody (Ab) could serve as novel immunotherapies for HCC. Multidrug resistance gene 2-deficient mice, a spontaneously occurring liver cancer model associated with aging, were administered rIL-2, rIL-18, and αPD-L1Ab. Antitumor effects were evaluated using computed tomography and serum alpha-fetoprotein levels. Significant tumor shrinkage was observed in the rIL-2+rIL-18+αPD-L1Ab group, but not in the rIL-2+αPD-L1Ab or rIL-18+αPD-L1Ab groups. Concurrent administration of αCD8-neutralizing antibody abolished the antitumor effect, indicating CD8^+^ T-cell dependency. Spatial gene expression profiling revealed that intratumoral CD8^+^ effector T cells express and secrete CCL5 after treatment, promoting CD8^+^ T-cell mobilization to the liver and enhancing antitumor efficacy. Pretreatment with a CCL5 neutralizing antibody suppressed CD8+ cell infiltration into the tumor, eliminating the antitumor effect. The triple combination therapy used in this study promotes the infiltration and maintenance of CD8^+^ T cells in the liver, suggesting a promising new immunotherapy for HCC.

Significance statement Although the emergence of immune checkpoint inhibitors has improved treatment outcomes for unresectable hepatocellular carcinoma, results remain insufficient. In this study, using a mouse model of spontaneously occurring liver cancer associated with aging, we demonstrated that combined therapy with interleukin (IL)-2, IL-18, and antiprogrammed cell death-ligand 1 antibody significantly reduced tumor size. This process required CD8^+^ T cells, which possessed effector memory T-cell functions and primarily recruited other CD8^+^ T cells via CCL5 production. These findings suggest that CD8^+^ T-cell infiltration is essential for limiting liver tumors and demonstrate that this triple combination therapy holds promise as a novel treatment.

## Introduction

Hepatocellular carcinoma (HCC) is the third leading cause of cancer-related mortality worldwide, with ∼700,000 new cases diagnosed in 2018, making it the third deadliest cancer globally (1). The prognosis remains poor, with a 5-year survival rate of only 13% for patients with unresectable or advanced HCC (2). Hepatitis B virus (HBV) and hepatitis C virus (HCV) infections are the most common causes of HCC (3). However, cases related to metabolic dysfunction-associated steatohepatitis (MASH) and alcohol consumption have increased in recent years (4). Current first-line treatments, such as atezolizumab–bevacizumab or tremelimumab–durvalumab, show limited efficacy, with response rates of ∼30% (5). Effective immunotherapy typically requires T-cell–mediated antitumor immunity; however, the lack of T-cell infiltration into the tumor microenvironment (TME) and the gradual functional impairment of antitumor T cells are major barriers in the case of HCC immunotherapy (6). Hence, new approaches are required to improve the immune microenvironment within the tumor and increase the efficacy of immunotherapy in patients with HCC.

Interleukin (IL)-18, an inflammatory cytokine that activates T and natural killer (NK) cells (7, 8), enhances interferon gamma (IFN-γ) production, thereby exerting antiviral and antitumor effects (9, 10). Ma et al. (11) reported that combining IL-18 with antiprogrammed death-1 (αPD-1) and anticytotoxic T-lymphocyte-associated protein 4 (αCTLA-4) antibodies (Abs) suppressed tumor growth in a CT-26 colon carcinoma model. Based on the findings from this report, we investigated the antitumor effects of combining recombinant IL-18 (rIL-18) with antiprogrammed death-ligand 1 Ab (αPD-L1Ab) in a multidrug resistance 2 gene knockout (*Mdr2-*KO) mouse-derived liver tumor model (12). However, the efficacy of this combination was insufficient as no tumor size reduction was observed. Given that IL-2 may synergize with IL-18 to enhance NK and CD8^+^ T-cell activity, we considered including IL-2 to potentiate the antitumor effect (13). Hence, in this study, we aimed to assess the efficacy of concomitant IL-2, IL-18, and αPD-L1 administration in treating liver tumors.

## Results

### Administration of rIL-2, rIL-18, and αPD-L1Ab suppresses liver tumor growth in Mdr2-KO mice

**Figure 1:**
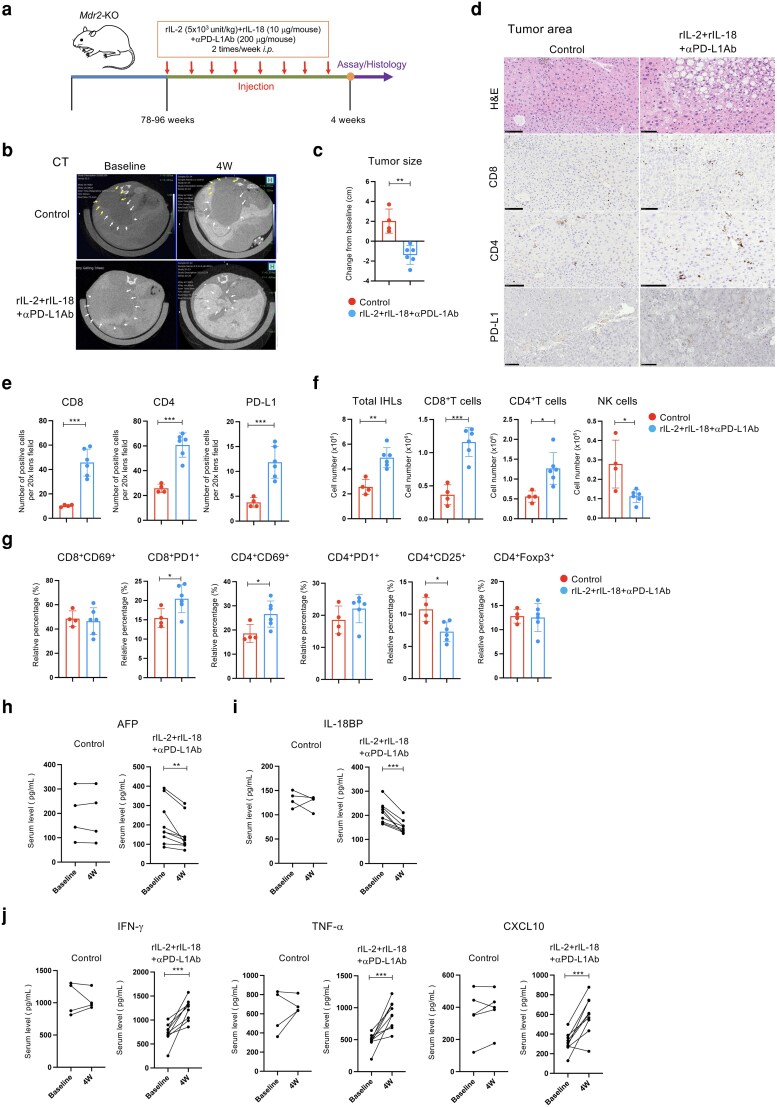
rIL-2+rIL-18+ αPD-L1 Ab suppresses liver tumor growth in *Mdr*2-KO mice. a) Experimental design. b) CT was performed 24 h later to assess tumor size and location in the liver. c) Comparison of tumor size before and after drug administration; the largest tumor was discernible on imaging. d) H&E and immunohistochemical staining. e) Quantification of positively stained cells in the liver. f, g) Immunohistochemical analysis of IHLs. h) Serum AFP, I) IL-18 binding protein (IL-18BP), J) IFN-γ, TNF-α, and CXCL10 levels were measured using an independent cohort (control *n* = 4, treatment *n* = 8) distinct from that used for the cellular analyses in (c) and (e–g). Data are presented as mean ± SD. * *P* < 0.05; ** *P* < 0.01; *** *P* < 0.005; **** *P* < 0.0001; n.s. not significant vs. the control-treated *Mdr2-*KO mice, as determined using Student's t test.

To assess the antitumor effects of the combination therapy, male *Mdr2-*KO mice (78–96 weeks old; *n* = 6) were treated twice weekly for 4 weeks with rIL-2 (5 × 10^3^ U/kg), rIL-18 (10 μg/mouse), and αPD-L1Ab (200 μg/mouse). Control mice (*n* = 4) received rat IgG2b injections (Fig. 1a). Forty-eight hours before the first administration, the mice were injected with a contrast agent (100 μL/mouse), and computed tomography (CT) imaging was performed to determine baseline tumor size and location. Tumor size increased in the control group compared with the pretreatment measurements (Fig. 1b). In contrast, mice that received triple therapy (rIL-2+rIL-18+αPD-L1Ab) exhibited a significant reduction in tumor size, demonstrating that the combination elicited potent antitumor effects (Fig. 1c).

Hematoxylin and eosin (H&E) staining of liver tissue revealed lymphocyte and mononuclear cell infiltration within and around tumors in control mice (Fig. 1d). In contrast, the triple therapy resulted in pronounced lymphocyte infiltration and increased apoptosis of the tumor cells (Fig. 1d). Immunostaining revealed a significant increase in the number of CD4^+^ and CD8^+^ T cells in the tumor region following combination therapy (Fig. 1d and e). Furthermore, PD-L1 staining showed an increase in mice treated with the triple therapy (Fig. 1d and e).

To evaluate immune cell dynamics, intrahepatic lymphocytes (IHLs) were isolated and analyzed. Triple therapy significantly increased total IHLs, CD4^+^ T cells, and CD8^+^ T cells, whereas NK cell counts were significantly decreased (Fig. 1f). In addition, CD8^+^PD-1^+^ and CD4^+^CD69^+^ T cells also increased. In contrast, the proportion of CD4^+^Foxp3^+^ T cells did not show significant alterations (Fig. 1g).

To assess systemic effects, alpha-fetoprotein (AFP), a liver tumor marker, was measured before and after triple therapy. AFP levels decreased significantly in the treated mice but remained unchanged in control mice (Fig. 1h). Moreover, the level of IL-18 binding protein (IL-18BP), which inhibits antitumor immunity (14, 15), decreased significantly following triple therapy (Fig. 1i). Serum levels of IFN-γ, TNF-α, and CXCL10 also increased significantly in the treated mice (Fig. 1j). Notably, the serum cytokine analyses were conducted using an independent cohort (control *n* = 4, treatment *n* = 8) from that used for the cellular analyses shown in Fig. 1c, e–g.

### Immunological analysis of IHLs in the early phase after administration of rIL-2, rIL-18, and αPD-L1Ab

**Figure 2:**
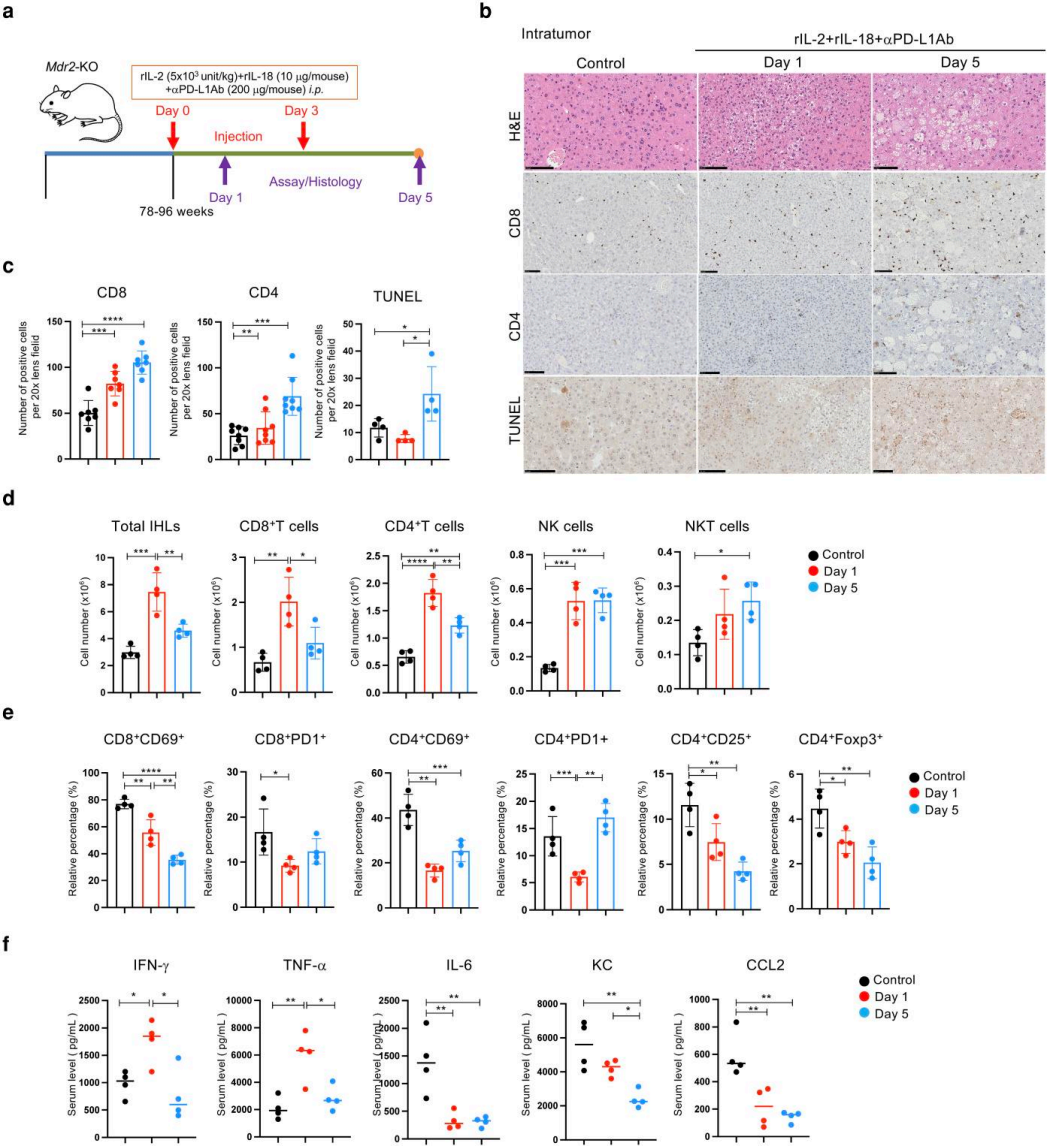
Changes were observed in early phase IHLs after administering rIL-2+rIL-18+αPD-L1Ab. a) Experimental design. b) H&E and immunohistochemical staining and TUNEL staining. c) Quantification of positively stained cells in the liver. d, e) Immunological analysis of IHLs. f) Serum cytokines and chemokines levels were measured using specimens from each group 4 weeks after treatment. Data are presented as mean ± SD. * *P* < 0.05; ** *P* < 0.01; *** *P* < 0.005; **** *P* < 0.0001, as determined using one-way ANOVA.

We next examined liver inflammatory cell dynamics histologically and immunologically at the early stages (days 1 and 5) after triple therapy (Fig. 2a). The analysis revealed significant infiltration of inflammatory cells into the tumor as early as 1 day posttreatment. Some tumors exhibited a foamy morphology, and, by day 5, certain tumors developed balloon-like (acidophilic body) structures, suggesting hepatocellular injury (Fig. 2b). Immunostaining showed a marked increase in the number of CD8^+^ and CD4^+^ T cells in the tumor during early stages of treatment (on days 1 and 5; Fig. 2b and c). TUNEL staining demonstrated a marked increase in TUNEL-positive hepatocytes on day 5 in the treated group compared with that in the control group (Fig. 2b and c).

The total number of IHLs peaked on day 1 but declined by day 5 (Fig. 2d). A similar trend was observed for CD4^+^ and CD8^+^ T cells. However, the proportion of NK cells (CD3^−^NK1.1^+^) peaked on day 5, whereas the total number of NK cells remained elevated on days 1 and 5. CD69 and PD-1 expression on CD4^+^ and CD8^+^ T cells decreased on day 1 compared with that in the control (Fig. 2e). Notably, the proportion of regulatory CD4^+^ T cells (CD4^+^CD25^+^ and CD4^+^Foxp3^+^) was significantly decreased on days 1 and 5 (Fig. 2e).

Serum cytokine and chemokine profiling revealed increased levels of IFN-γ and tumor necrosis factor-alpha (TNF-α) on day 1, which returned to baseline by day 5. Conversely, levels of IL-6, keratinocyte chemoattractant (KC), and CCL2 decreased after treatment (Fig. 2f).

### Administration of rIL-2, rIL-18, and αPD-L1Ab is required for antitumor effects

**Figure 3:**
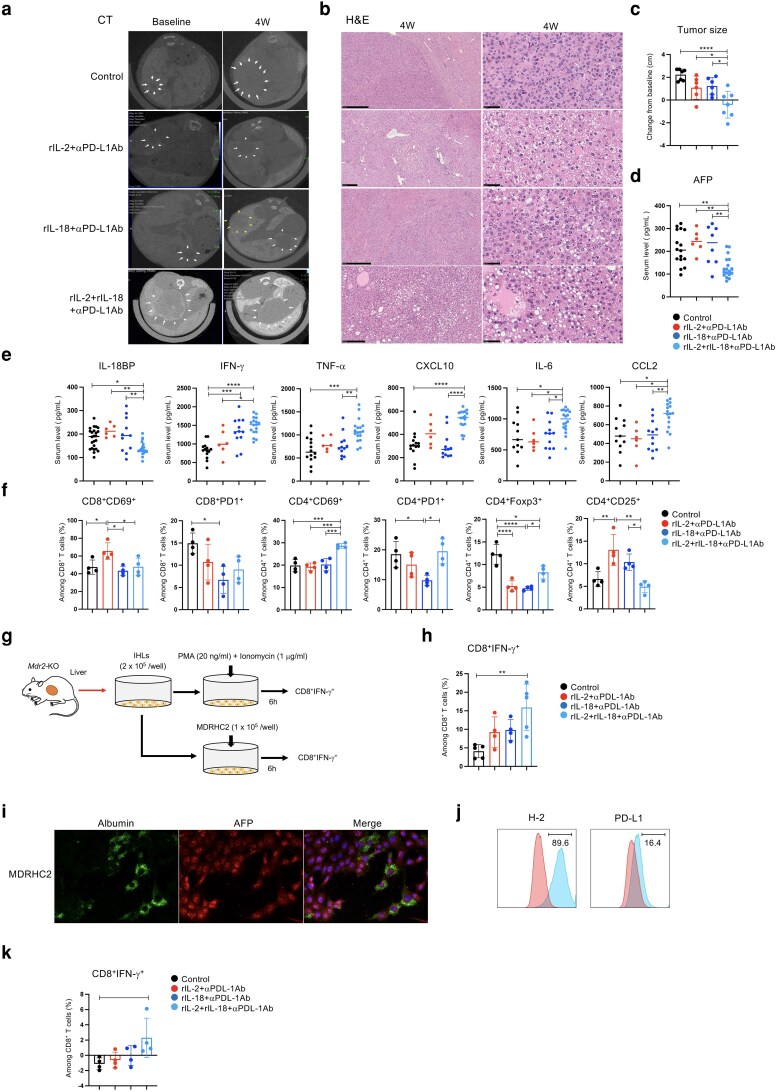
Administration of triple therapy (rIL-2+rIL-18+αPD-L1Ab) elicits desirable antitumor effects in the liver. a) CT was performed 24 h later to assess tumor size and location in the liver. b) Liver histology analysis using H&E staining after treatment. c) Comparison of tumor size before and after treatment, with the largest tumor discernible on imaging. d, e) Serum AFP, cytokines, and chemokines were measured using specimens from each group 4 weeks after treatment. Data represent pooled biological replicates from the tumor assessment cohort (b, c) and additional independent experiments. Total sample sizes: control (*n* = 14), rIL-2+αPD-L1Ab (*n* = 6), rIL-18+αPD-L1Ab (*n* = 12), and triple therapy (*n* = 18). f) IHLs were analyzed to examine the host immune response against liver tumors. g, h) The production capacity of CD8^+^ T-cell-derived IFN-γ was analyzed. i) Single-color and merged images depicting immunofluorescence results from MDRHC2 cell lines. j) Expression of H-2 and PD-L1 in MDRHC2 cells. k) Percentage of IFN-γ-producing positive cells on CD8^+^ T cells in MDRHC2 cell lines. Data are presented as mean ± SD. * *P* < 0.05; ** *P* < 0.01; *** *P* < 0.005; **** *P* < 0.0001, as determined using one-way ANOVA.

To evaluate the antitumor effects of the triple therapy, *Mdr2*-KO mice (78–96 weeks old) were intraperitoneally administered with rat IgG2b (control, *n* = 7), rIL-2 (5 × 10^3^ unit/kg) + αPD-L1Ab (200 mg/mouse; *n* = 6), rIL-18 (10 mg/mouse) + αPD-L1Ab (200 mg/mouse; *n* = 6), or rIL-2+rIL-18+αPD-L1Ab (*n* = 7) twice weekly for 4 weeks. Tumor assessments revealed that administration of rat IgG2b, rIL-2+αPD-L1Ab, or rIL-18+αPD-L1Ab resulted in increased tumor size. In contrast, tumor size decreased significantly after triple therapy compared with either the control or rIL-18+αPD-L1Ab-treated groups (Fig. 3a and c).

Histological analysis of the liver tissue revealed distinct tumor hollowing in the rIL-2+αPD-L1Ab and the triple therapy groups, a feature absent in the control group. Additionally, tumor cell apoptosis and inflammatory cell infiltration were observed following triple therapy (Fig. 3b).

For serum biomarker and cytokine analyses, data from the cohort used for tumor assessment (Fig. 3b and c) were combined with those from additional independent experiments performed at different times to increase the number of biological replicates. Therefore, the data shown in Fig. 3d and e represent pooled biological replicates rather than technical replicates, and each data point corresponds to an individual mouse.

Serum AFP levels were significantly reduced in the triple therapy group compared with those in the control or rIL-18+αPD-L1Ab-treated groups (Fig. 3d). In addition, a significant reduction in IL-18BP was observed in the triple therapy-administered mice compared with that in the control or other treatment groups (Fig. 3e).

IFN-γ, IL-6, and CCL2 levels were significantly higher in the triple therapy group than in the control or rIL-2+αPD-L1Ab groups. In contrast, TNF-α and CXCL10 levels were significantly increased compared with those in the rIL-18+αPD-L1Ab or control groups (Fig. 3e). Additional analysis revealed elevated IL-2, CXCL1, CCL3, and CCL4 levels in the triple therapy group (Fig. 1  S1b). However, no significant differences in serum alanine aminotransferase (ALT) levels were observed between treatment groups (Fig. 1  S1a).

To further investigate immune responses against liver tumors, IHL analysis was conducted, which revealed that CD8^+^CD69^+^ T cells were significantly increased in the rIL-2+αPD-L1Ab group compared with those in the control or other treatment groups (Fig. 3f). Conversely, the proportion of CD8^+^PD-1^+^ T cells was significantly lower in the rIL-18+αPD-L1Ab group than in the control group (Fig. 3f).

The proportion of CD4^+^CD69^+^ T cells increased significantly in the triple therapy group compared with the double combination therapy and control groups. CD4^+^Foxp3^+^ T cells were significantly decreased in all treatment groups compared to the control group (Fig. 3f).

### Cytokine production and tumor-specific response of CD8^+^ T cells

To assess cytokine production capacity, we analyzed IFN-γ production by CD8^+^ T cells in liver lymphocytes following triple therapy. Lymphocytes were stimulated ex vivo with phorbol 12-myristate 13-acetate (PMA) and ionomycin, and IFN-γ production was significantly higher in the triple therapy group than in the control group (Fig. 3g and h).

Identifying tumor-specific CD8^+^ T cells is challenging in the liver tumor model used in our study because of the *Mdr2*-KO genetic background, which lacks known tumor antigens or AFP epitopes (16). To overcome this limitation, we attempted to establish tumor cell lines from liver tumors of *Mdr2*-KO mice. As shown in Fig. 3i, we successfully generated the albumin-positive, AFP-positive, CK-19-negative MDRHC2 cell line from the liver tumor tissue. The MDRHC2 cells were strongly H-2-positive and expressed high levels of PD-L1 (Fig. 3j), confirming their tumorigenic and immunogenic properties. This cell line was subsequently used as a target to detect tumor-specific responses of CD8^+^ T cells as described below.

Mice were treated with rIL-2+αPD-L1Ab (*n* = 4), rIL-18+αPD-L1Ab (*n* = 4), or rIL-2+rIL-18+αPD-L1Ab (*n* = 4) twice weekly for 4 weeks; thereafter, lymphocytes from the spleen were isolated. These lymphocytes (5 × 10^5^/well) were then cocultured with MDRHC2 cells (1 × 10^5^/well) for 4 h, and IFN-γ production by CD8^+^ T cells was analyzed.

**Figure 4:**
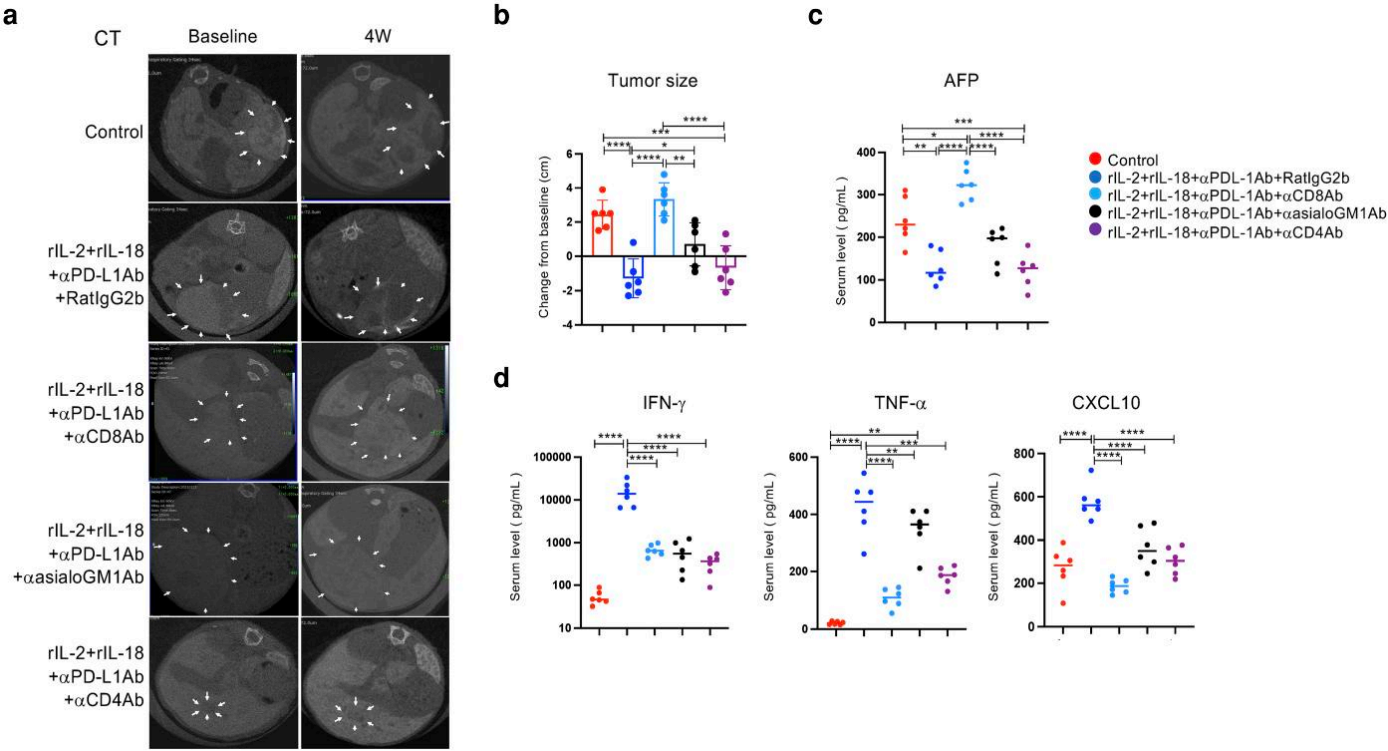
CD8^+^ T cells are essential for the antitumor effects of rIL2+rIL18+αPD-L1Ab therapy. a) CT was performed 24 h later to assess tumor size and location in the liver. b) Comparison of tumor size before and after treatment, with the largest tumor discernible on imaging. c, d) Serum AFP, cytokines, and chemokines were measured using specimens from each group 4 weeks after treatment. Data are presented as mean ± SD. * *P* < 0.05; ** *P* < 0.01; *** *P* < 0.005; **** *P* < 0.0001, as determined using one-way ANOVA.

The number of IFN-γ-producing CD8^+^ T cells from the spleen was significantly increased in the triple therapy group, with a moderate increase observed in the rIL-2+αPD-L1Ab group (Fig. 4k). However, analysis of splenic lymphocytes revealed a significant increase in IFN-γ-producing CD8^+^ T cells only in the triple therapy group, suggesting that this treatment uniquely enhances tumor-specific responses.

### CD8^+^ T cells are required for the antitumor effects of triple administration of rIL-2, rIL-18, and αPD-L1Ab

To identify the effector cells essential for the antitumor effects of the combination therapy, we assessed the tumor size using anti-CD8-neutralizing Ab (αCD8Ab), antiasialoGM1Ab (αasialoGM1Ab), and anti-CD4-neutralizing Ab (αCD4Ab). Male *Mdr2*-KO mice (78–96 weeks old) were treated intraperitoneally twice a week for 4 weeks with either rat IgG2b (*n* = 6) or rIL-2+rIL-18+αPD-L1Ab+rat IgG2b (*n* = 6) coadministered with αCD8Ab (*n* = 6), αasialoGM1Ab (*n* = 6), or αCD4Ab (*n* = 6). αCD8Ab, αCD4Ab, and αasialoGM1Ab were administered intraperitoneally twice a week for 4 weeks. After 4 weeks, mice that received triple therapy + rat IgG2b exhibited a trend toward reduced tumor size (Fig. 4a and b). However, tumor size increased significantly only in the triple therapy + αCD8Ab-treated group compared with the rat IgG2b, αasialoGM1Ab, or αCD4Ab groups. This finding indicates that CD8^+^ T cells are indispensable for the antitumor effects of the triple therapy. The role of CD8^+^ T cells was further substantiated by the observation that serum AFP levels did not decrease following CD8^+^ T-cell depletion. In contrast, AFP levels were significantly reduced in mice treated with triple therapy + rat IgG2b, αasialoGM1Ab, or αCD4Ab compared with that in control mice (Fig. 4c). To assess the impact of immune cell depletion on inflammatory responses, we measured serum cytokine and chemokine levels. IFN-γ, TNF-α, and CXCL10 levels were significantly reduced in the triple therapy + αCD8Ab, αasialoGM1Ab, and αCD4Ab groups compared with those in the triple therapy + rat IgG2b group (Fig. 4d).

### Xenium spatial transcriptomics reveals therapy-induced CD8^+^ T-cell reprogramming

**Figure 5:**
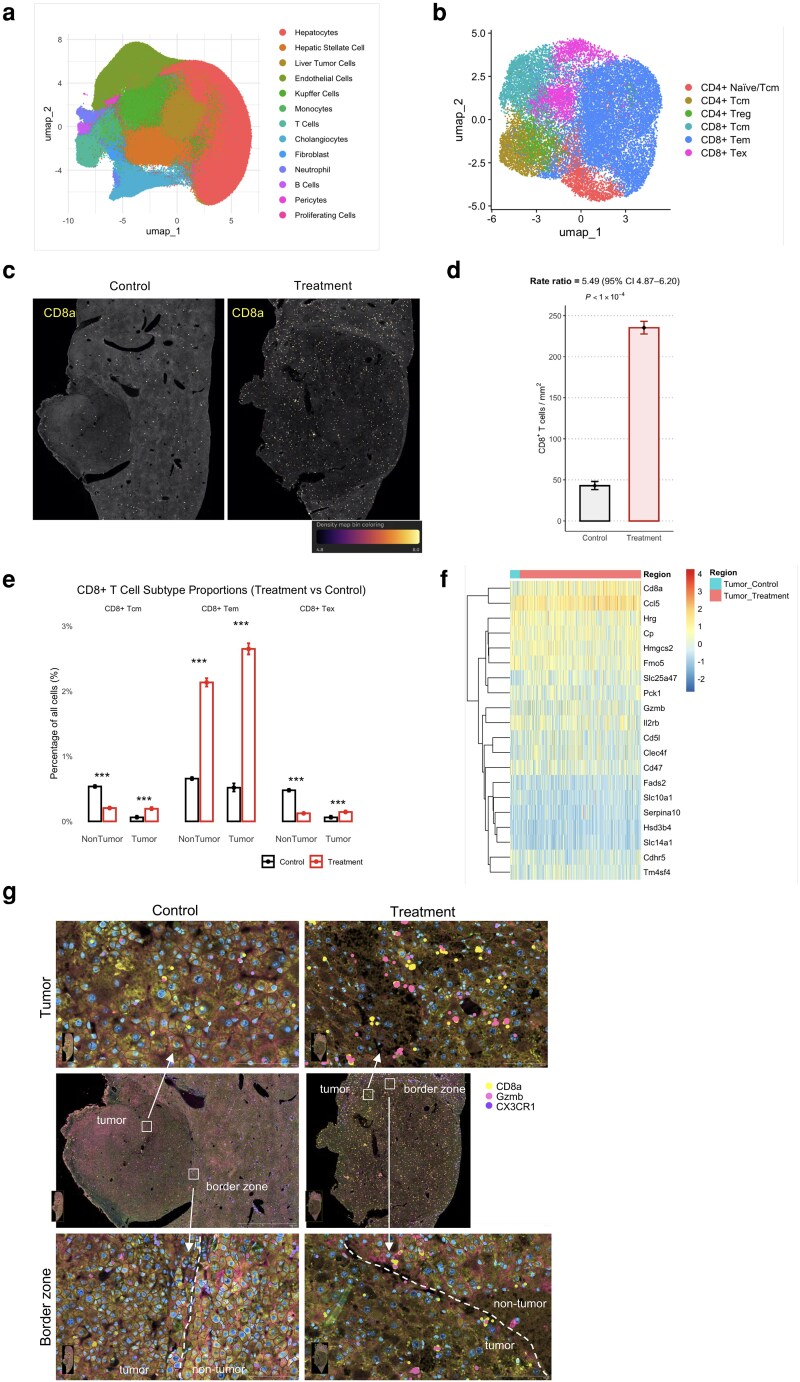
Xenium spatial transcriptomics reveal CD8^+^ T-cell remodeling following triple therapy. a) UMAP embedding of 834,915 cells derived from the four ROIs. b) After renormalization with SCTransform, the subset was reclustered using the Leiden algorithm (resolution = 0.8; 13 clusters) and visualized in a high-resolution UMAP. c) Spatial quantification of tumor-infiltrating CD8^+^ T cells. d) Comparison of CD8^+^ T-cell density in tumor ROIs [cells mm^−2^; mean ± exact Poisson 95% CI]. e) Proportional enrichment of CD8^+^ T-cell subsets after triple therapy. Bar plots display the percentage of all Xenium-profiled cells that were classified as CD8^+^ Tcm, Tem, or Tex cells in the nontumor (left) and tumor (right) regions. Bars show mean proportion and λ-adjusted Wilson 95% CI (error bars). Asterisks indicate Fisher's exact test comparing treatment with control for each subset within each compartment (*P < 0.05; **P < 0.01; ***P < 0.001). f) Scaled-expression heat map of the top 20 upregulated genes in tumor-infiltrating CD8^+^ T cells. G) Spatial distribution of CD8^+^ T-cell markers in liver tumors.

Flow cytometry results showed that triple therapy expands hepatic CD8^+^ T cells; we investigated their spatial organization using Xenium spatial transcriptomics. Four regions of interest (ROIs), representing tumor from control (vehicle-treated) mice, tumor from treated (administered with triple therapy) mice, adjacent nontumor liver from control mice, and adjacent nontumor liver from treated mice, were selected based on H&E-guided analysis. The results yielded 834,915 single-cell transcriptomes. SCTransform normalization and Leiden clustering (resolution 0.6) resolved 18 distinct clusters corresponding to parenchymal, stromal, and immune compartments in the uniform manifold approximation and projection (UMAP) projection. Hepatocytes, endothelial and stellate cells, cholangiocytes, Kupffer cells, liver tumor cells, and multiple immune lineages (Fig. 5a) were identified and annotated based on the presence of canonical markers, confirming the preservation of native tissue complexity.

To focus on lymphocytes, we extracted 21,857 cells expressing more than three counts of *Cd4*, *Cd8a*, or *Cd3d*, renormalized, and reclustered (resolution 0.8) into 13 groups, consolidated into six functional T-cell states: CD4^+^ naïve/Tcm, CD4^+^ Tcm, CD4^+^ regulatory T cells, CD8^+^ Tcm, CD8^+^ Tem, and CD8^+^ exhausted T cells (Tex cells) (Fig. 5b). Whole-section Xenium imaging showed a sparse *Cd8a^+^* signal in control tumors but dense, widespread signal after therapy (Fig. 5c). Quantitatively, CD8a^+^ cell density increased from 42.9 mm^−2^ (297 cells in 6.93 mm^2^) to 235.3 mm^−2^ (3,697 cells in 15.71 mm^2^), a 5.49-fold rise (95% CI: 4.87–6.20, *P* < 1 × 10^−4^; Fig. 5d). Subset analysis revealed significant enrichment of intratumoral CD8^+^ Tcm (∼4-fold), Tem (>5-fold; ∼3% of all cells), and Tex (∼2-fold), with selective Tem expansion observed in nontumor tissues (Fig. 5e).

Top upregulated genes included *Cd8a* (log_2_FC: 1.39, FDR: 2.4 × 10^−36^); the chemokine *Ccl5* (0.51, 6.4 × 10^−7^); cytotoxic/metabolic regulators *Gzmb* (0.50), *Hmgcs2* (0.73), and *Slc25a47* (1.00); immune modulator *Cd5l* (1.21); and scavenger receptor *Clec4f* (1.12). Heatmap visualization (Fig. 5f) of the top-20 induced genes highlighted a coherent transcriptional module comprising cytotoxic (*Cd8a*, *Gzmb*), chemokine (*Ccl5*), metabolic (*Hmgcs2*, *Slc25a47*), and phagocytic programs (*Cd5l, Clec4f*).

High-resolution overlays of *Cd8a*, *Gzmb*, and *Cx3cr1* transcripts revealed triple-positive lymphocyte clusters forming dense microaggregates within tumor cores and rims after treatment—structures absent in control sections (Fig. 5g). These results demonstrate that triple therapy enhances CD8^+^ T-cell recruitment, as well as skews them toward a cytotoxic Tem-dominated phenotype spatially distributed throughout the TME.

### CCL5+ CD8+ Tem cells increase in liver tumors after triple therapy

**Figure 6:**
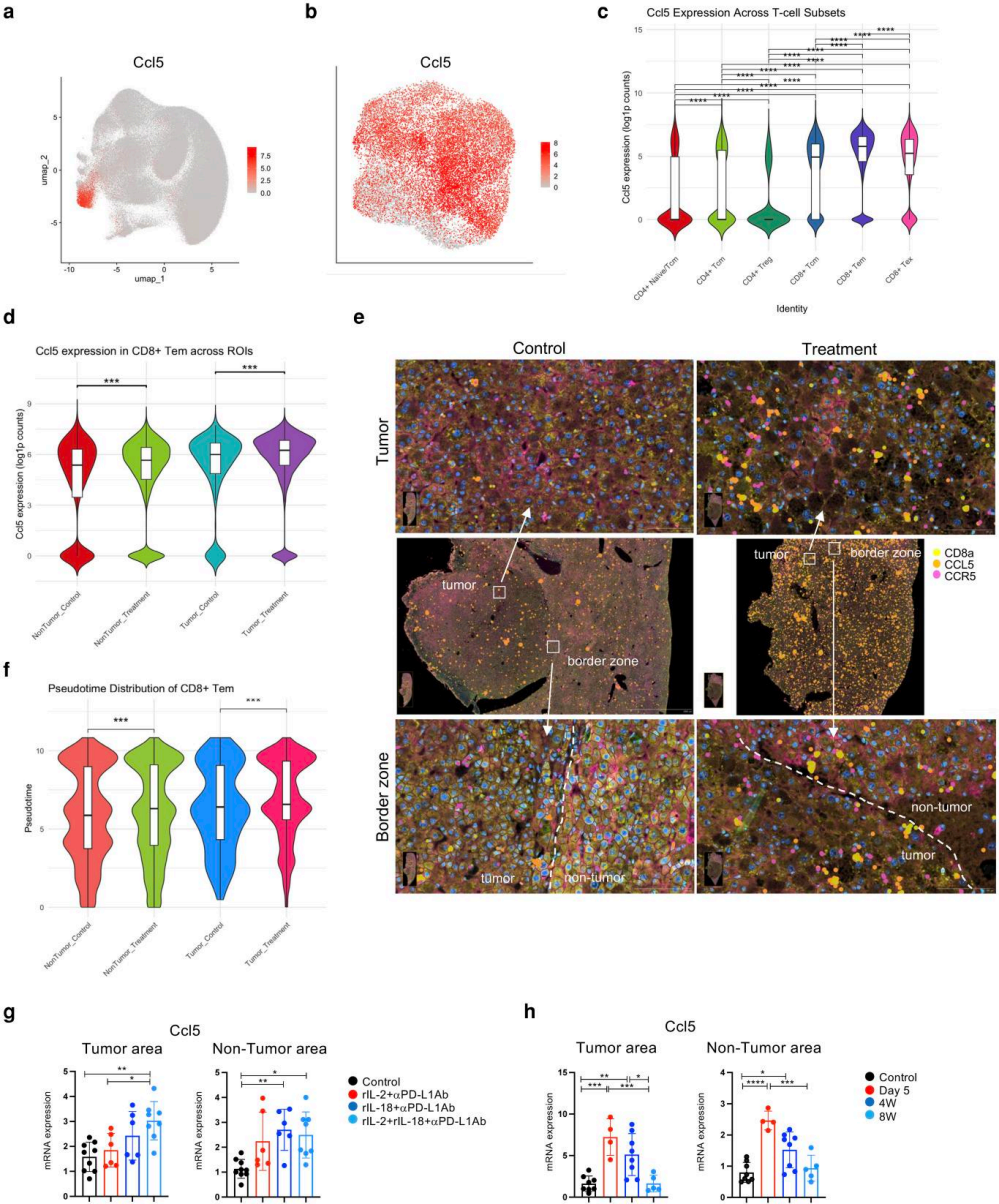
CCL5^+^CD8^+^ Tem cells are induced and maintained in tumors after triple therapy. a) UMAP localization of *Ccl5*-expressing T cells in the integrated Xenium dataset. b) Ccl5 expression was highest in the CD8^+^ Tem cluster. c) *Ccl5* is most abundantly expressed in the CD8^+^ Tem subset. Horizontal brackets indicate pairwise Wilcoxon rank-sum tests with Bonferroni correction; **** *P* < 0.0001, *** *P* < 0.001, ** *P* < 0.01, * *P* < 0.05. d) *Ccl5* expression in CD8^+^ Tem cells across spatial ROIs. Violin plots (with internal box-and-whisker overlays) show single-cell *Ccl5* expression (log_1_*p*-normalized counts) in CD8^+^ Tem cells isolated from four Xenium ROIs. Horizontal brackets indicate within-region comparisons (two-sided Wilcoxon rank-sum test, Bonferroni corrected); *** *P* < 0.001. e) Spatial colocalization of *Ccl5-expressing* CD8^+^ T cells in liver tumors. f) Pseudotime dynamics of CD8^+^ Tem cells in tumor and nontumor regions. Violin plots display the distribution of pseudotime values in CD8^+^ Tem cells across the four ROIs. Each violin plot is overlaid with a boxplot, and statistical comparisons were conducted using Wilcoxon tests. g, h) mRNA expression of *Ccl5* in the liver as determined by RT-qPCR. Data represent the mean ± SD. * *P* < 0.05; ** *P* < 0.01; *** *P* < 0.005; determined using one-way ANOVA.

Projection of *Ccl5* onto the integrated UMAP (Fig. 6a) revealed expression confined to the T-cell compartment, with strongest signal in the CD8^+^ Tem cluster (Fig. 6b). Among all 21,857 T cells, Tem cells displayed the highest *Ccl5* expression. Pairwise Wilcoxon tests confirmed significant expression differences between Tem and all other subsets (*P* < 10^−4^; Fig. 6c). Therapy significantly increased *Ccl5* levels in both tumor and nontumor ROIs (**P < 0.001; Fig. 6d), indicating a broad induction of the *Ccl5* program.

Xenium imaging revealed clusters of *Cd8a^+^Ccl5^+^Ccr5^+^* lymphocytes forming belts along the tumor–liver interface and projecting into the tumor core—patterns absent in controls (Fig. 6e). In control tumors, CD8a^+^ cells are scattered and rarely coexpress CCL5 or CCR5. In contrast, triple therapy generates dense clusters of CD8a^+^ cells that coexpress both CCL5 and its receptor CCR5 across the tumor mass, with particularly high concentrations at the invasive margin and a clear enrichment within the tumor core.

Pseudotime analysis revealed that, following therapy, CD8^+^ Tem cells exhibited an advanced state of differentiation in both tumor and nontumor regions (*P* < 0.001; Fig. 6f). As described above, triple therapy robustly increased *Ccl5*^+^CD8^+^ Tem cells within tumors. To evaluate how *Ccl5* expression responds to different agent combinations, RNA was extracted from tumor and nontumor tissues and analyzed using RT-PCR. In tumors, *Ccl5* expression was significantly elevated in the triple therapy group compared with that in the control or rIL-2+αPD-L1 groups (Fig. 6g). In nontumor tissue, *Ccl5* levels were significantly increased in the rIL-18+αPD-L1Ab and triple therapy groups compared with the control. Temporal expression analysis revealed that *Ccl5* expression peaked 5 days posttreatment in both tumor and nontumor regions and declined by weeks 4 and 8 (Fig. 6h).

### CCL5 is essential for the antitumor effect of triple administration with rIL-2, rIL-18, and αPD-L1Ab

**Figure 7:**
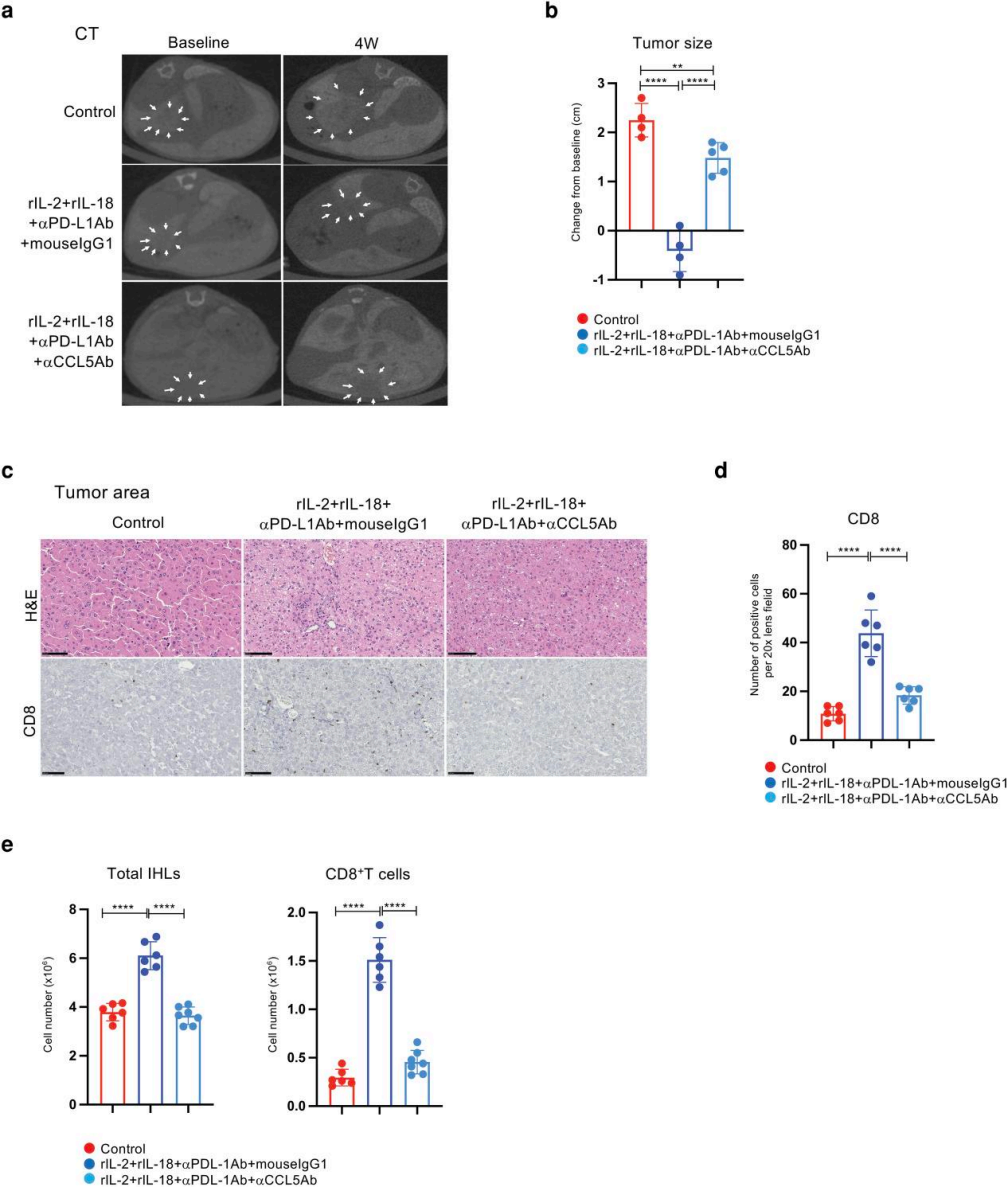
CCL5 is essential for the antitumor effects of rIL2+rIL18+αPD-L1Ab therapy. a) CT was performed 24 h later to assess tumor size and location in the liver. b) Comparison of tumor size before and after treatment, with the largest tumor discernible on imaging. c) H&E and immunohistochemical staining. d) Quantification of positively stained (CD8^+^) T cells in the liver. e, f) Cell number of total IHLs and CD8^+^ T cells in the liver. Data are presented as mean ± SD. * *P* < 0.05; ** *P* < 0.01; *** *P* < 0.005; **** *P* < 0.0001, as determined using one-way ANOVA.

Results from Xenium spatial transcriptomics analysis revealed a marked increase in CCL5^+^CD8^+^ T cells within liver tumors after triple therapy administration. To examine the impact of CCL5 on antitumor effects, tumor size was evaluated using an anti-CCL5 neutralizing antibody (αCCL5Ab). Male *Mdr2*-deficient mice (78–96 weeks old) received intraperitoneal injections twice weekly for 4 weeks. Groups included: control (*n* = 4), rIL-2+rIL-18+αPD-L1Ab+mouse gG1 treatment group (*n* = 4), rIL-2+rIL-18+αPD-L1Ab+αCCL5Ab (200 μg/mouse) treatment group (*n* = 5). αCCL5Ab was administered intraperitoneally twice weekly starting the day before triple therapy. After 4 weeks, a trend toward tumor size reduction was observed in the triple therapy + mouse IgG1 group (Fig. 7a and b). However, tumor size significantly increased in the triple therapy + αCCL5Ab group (Fig. 7a and b). This finding indicates that CCL5 is essential for the antitumor effect of the triple therapy. Immunohistochemical analysis of CD8 T-cell numbers within tumors revealed that the increase in CD8-positive cells observed in the triple therapy group was significantly reduced in the αCCL5Ab group (Fig. 7c and d). Similarly, measurements of total lymphocyte numbers and CD8-positive T-cell numbers in the liver yielded comparable results. These findings demonstrate that triple therapy induces CCL5^+^CD8^+^ T cells, and that this CCL5 is involved in the migration and antitumor effects of other CD8 T cells.

## Discussion

In this study, we demonstrated that triple therapy comprising rIL-2, rIL-18, and αPD-L1Ab significantly reduced tumor burden and exerted robust antitumor effects in a liver tumor model derived from *Mdr2*-KO mice. This antitumor effect was not observed with rIL-2+ αPD-L1Ab or rIL-18+ αPD-L1Ab alone. Cell depletion experiments confirmed that CD8^+^ T cells are essential for tumor suppression. Immunological analysis revealed a significant increase in CD8^+^ T cells in both tumor and nontumor liver regions following triple therapy. Spatial transcriptomics further showed that treatment induced infiltration of CCL5^+^CD8^+^ Tem cells in both compartments.

In addition to CD8^+^ T-cell expansion, we observed distinct temporal changes in intrahepatic innate and adaptive immune populations by flow cytometric analysis. Intrahepatic NK cells (defined as CD3^−^NK1.1^+^ cells) showed rapid early expansion, peaking at days 1 and 5 after treatment, followed by contraction at week 4. In parallel, serum IFN-γ levels increased on day 1, decreased on day 5, and rose again on week 4. Intrahepatic CD8^+^ T cells displayed a biphasic pattern, with an initial increase on day 1, transient decrease on day 5, and subsequent increase on week 4.

This kinetic pattern is consistent with the distinct temporal roles of innate and adaptive immune responses. NK cells respond rapidly to cytokine stimulation, particularly IL-2 and IL-18, leading to early activation and IFN-γ production within days. However, NK responses are typically transient and may contract over time due to redistribution or functional exhaustion (17–19). In contrast, CD8^+^ T cells require antigen priming and clonal expansion but can generate sustained antitumor immunity (20, 21). The delayed increase in intrahepatic CD8^+^ T cells at week 4, together with the re-elevation of IFN-γ at this time point, may reflect the establishment of a CD8^+^ T-cell–mediated adaptive immune response following the initial NK-driven inflammatory phase. Early NK-derived IFN-γ may help shape the intrahepatic immune microenvironment and facilitate subsequent CD8^+^ T-cell priming (19). This temporal transition from NK-dominated early responses to CD8^+^ T-cell–mediated immunity may partly explain the progressive tumor regression observed over time.

Cholestasis induces hepatocellular injury in liver tumor models, leading to fibrosis-driven tumor development (22, 23). The *Mdr2-*KO mouse model recapitulates key pathogenic features of human HCC. Persistent liver inflammation triggers cycles of damage and repair, resulting in genetic abnormalities in hepatocytes and excessive extracellular matrix deposition by hepatic stellate cells, culminating in fibrosis and HCC (12). This model also exhibits elevated AFP levels, a clinical hallmark of HCC, supporting its translational relevance. Despite the long latency (∼1 year) for tumor development, the model recapitulates tumor-associated immune tolerance and is valuable for evaluating novel immunotherapies.

Triple therapy resulted in increased CD8^+^ Tem cells, particularly the CCL5^+^ subset, in liver tumors. Solid tumors can produce chemokines, such as CCL5, to recruit CD8^+^ T cells and elicit antitumor responses (24). Additionally, IL-18–activated NK cells can recruit immature dendritic cells via CCR5 in a CCL5-dependent manner, leading to enhanced CD8^+^ T-cell infiltration (25).

In our study, however, CCL5 expression was detected exclusively in CD8^+^ T cells and not in tumor cells, macrophages, or monocytes. These findings suggest that triple therapy promotes CCL5 production by CD8^+^ T cells rather than by myeloid or tumor-derived sources.

In human colorectal cancer liver metastases, CD8^+^ T cells at the tumor margin produce CCL5; however, this has been linked to tumor-promoting effects by modulating tumor-associated macrophages. Blocking the CCR5–CCL5 axis in such contexts has shown therapeutic potential (26). In contrast, to our knowledge, no prior reports have indicated that CCL5^+^CD8^+^ T cells possess effector functions and directly contribute to antitumor immunity.

Combined administration of rIL-2 and rIL-18 without αPD-L1Ab failed to induce tumor regression (Fig. S2), indicating that checkpoint blockade is essential for therapeutic efficacy. αPD-L1Ab reinvigorates dysfunctional tumor antigen-specific T cells (27), suggesting that its contribution lies in restoring CD8^+^ T-cell function. Similarly, neither rIL-2+αPD-L1Ab nor rIL-18+αPD-L1Ab was sufficient to suppress tumor growth. These results emphasize the necessity of concurrent administration of all three agents. The observed increase in *Ccl5* mRNA in both tumor and nontumor regions strongly correlated with therapeutic efficacy. Together, our findings indicate that the antitumor effect of triple therapy depends on the expansion and functional reprogramming of CCL5^+^CD8^+^ T cells.

Although tumor-specific CD8^+^ T cells clearly contribute to the observed antitumor effect, our data suggest that both antigen-specific and generalized immune activation mechanisms may be involved. Functional assays using splenic CD8^+^ T cells demonstrated enhanced IFN-γ production in response to the MDRHC2 tumor cell line, supporting the induction of tumor-reactive T cells at the systemic level. Simultaneously, spatial transcriptomic analyses revealed broad recruitment of CD8^+^ T cells into both tumor and nontumor liver regions, likely driven in part by CCL5-mediated chemotactic signals. These findings suggest that cytokine priming promotes not only tumor-specific responses but also a generalized activation and redistribution of CD8^+^ T cells that facilitate intratumoral infiltration and maintenance. Thus, both tumor-specific reactivity and chemokine-driven T-cell recruitment likely cooperate to mediate the overall therapeutic effect.

Xenium spatial transcriptomics revealed how rIL-2+rIL-18+αPD-L1Ab therapy reshapes the liver TME, offering a high-resolution mechanistic blueprint. Integration of over 830,000 single-cell transcriptomes across defined ROIs in treated and control mice showed that this therapy increased the density of tumor-infiltrating CD8^+^ T cells by 5- to 6-fold and redirected them toward the Tem phenotype. Spatially, *Cd8a^+^* Tem cells formed dense bands along the tumor–liver interface and penetrated the tumor core—patterns rarely seen in untreated tumors. Mechanistically, triple therapy selectively and persistently induced *Ccl5* expression in CD8^+^ Tem cells. Trajectory modeling using tradeSeq showed that *Ccl5* expression diverged from baseline early in pseudotime, preceding terminal differentiation, suggesting that this treatment actively reprograms chemotactic signaling rather than merely amplifying baseline activity. This finding aligns with those from previous studies (28), demonstrating that memory-like CD8^+^ T cells secrete CCL5 to promote their own motility and survival via an autocrine CCR5 loop, and is further supported by single-cell data showing CCL5-high Tem subsets in human (29). High-resolution overlays confirmed colocalization of Ccl5–Ccr5, reinforcing the existence of an autocrine/paracrine loop that stabilizes CD8^+^ Tem clusters and enhances cytolytic activity. The effector profile induced by the triple therapy is multifaceted: beyond changes in *Ccl5* expression, coordinated upregulation of *Gzmb*, *Hmgcs2*, *Slc25a47*, and scavenger receptors (*Cd5l*, *Clec4f*) was observed, implicating a broad program involving metabolic rewiring and enhanced phagocytic–cytotoxic coupling. This transcriptional profile likely underlies the potent antitumor activity of CD8^+^ Tem cells, which possess both rapid reactivation capacity and sustained effector functions.

rIL-2 and rIL-18 synergistically enhance IFN-γ production and cytotoxicity in vivo (9, 30). However, exhausted CD8^+^ T cells often exhibit reduced IL-18Rα expression, limiting their responsiveness (31). In the current study, PD-L1 blockade may restore this responsiveness, enabling full activation of the CCL5 axis. Spatial transcriptomic analysis further revealed increased Il18r1 expression in CD8^+^ effector T cells in both tumor and nontumor regions in treated mice, suggesting enhanced responsiveness to IL-18 signaling and supporting an IL-18–dependent mechanism underlying CD8^+^ T-cell activation (Fig. S3).

Clinically, these findings highlight CCL5-high CD8^+^ Tem cells as candidate pharmacodynamic biomarkers and potential therapeutic targets. The spatial density of these cells, quantifiable by Xenium, could be used to stratify responders in trials of IL-2– or IL-18–based combination therapies. Furthermore, rational augmentation of the CCL5–CCR5 axis—via local chemokine delivery, CCR5-engineered T cells, or modulation of the hepatic niche [e.g. through CCL5-mediated fibrosis resolution by Trm cells (32)]—may enhance therapeutic outcomes. To assess the long-term efficacy of the formulated triple therapy, we extended treatment to 8 weeks, which led to significant tumor growth suppression compared with that in the control group (Fig. S4). Comparable antitumor effects were also observed with αPD-1Ab (Fig. S5), further supporting the translational potential of this combination approach.

Nevertheless, a few limitations merit consideration. The *Mdr2*-KO model represents cholestasis-driven liver injury progressing to fibrosis and hepatocarcinogenesis and therefore does not fully recapitulate viral or MASH-associated HCC. However, inflammation-driven tumor development is a shared pathogenic mechanism across multiple etiologies of human HCC, supporting the biological relevance of this model for studying immune–tumor interactions. First, we used a single mouse model of HCC and a single posttreatment time point. Temporal sampling and validation in orthogonal models or using patient biopsies are required to determine whether CCL5-centered reprogramming is universal or situation-dependent. Second, the Xenium platform measures steady-state mRNA levels; establishing causality will require complementary proteomic or functional assays (e.g. CCR5 blockade and CCL5 neutralization). Third, although both IL-2 and IL-18 promote effector cell differentiation, elucidating their individual and synergistic contributions to the CCL5 program, particularly under checkpoint blockade, must still be analyzed.

These findings provide a conceptual framework for converting “immune-cold” liver tumors into immune-infiltrated, therapy-responsive lesions.

In conclusion, triple therapy using rIL-2, rIL-18, and αPD-L1Ab represents a distinct immunotherapeutic strategy with a mechanism of action distinct from that of conventional HCC therapies. The approach significantly inhibited tumor growth in a liver tumor model derived from *Mdr2*-KO mice. This effect was mediated by CD8^+^ T-cell–driven immune responses, including the recruitment and functional reprogramming of CCL5^+^ effector memory subsets. Our findings offer mechanistic insight and clinical rationale for further development of this combination strategy as a novel immunotherapeutic approach for HCC.

## Materials and methods

### Ethics statement

All animal experiments were conducted in accordance with the institutional guidelines of the National Academy of Sciences (Guide for the Care and Use of Laboratory Animals). The study protocol was approved by the Research Committee of the Tokyo Metropolitan Komagome Hospital (approval number 2). This study was conducted in compliance with the ARRIVE guidelines for reporting in vivo experiments.

### Animals and treatments

All mice were housed in ventilated cages under 12 h light/dark cycles with free access to enrichment, water, and standard feed. Male multidrug resistance-2 gene knockout (*Mdr2-*KO) mice (FVB.129P2-Abcb4tm1Bor/J, #002539) were purchased from Jackson Laboratory (Bar Harbor, ME, USA). At 78–96 weeks of age, mice were randomly assigned to different treatment groups and received intraperitoneal injections of the designated agents.

### Reagents and treatments

Recombinant mouse IL-18 was provided by Dr. Tanaka (Nagasaki University), and recombinant human IL-2 was purchased from Shionogi & Co., Ltd. (Osaka, Japan). For in vivo administration, mice received intraperitoneal injections of 200 μg antimouse PD-L1Ab or rat IgG2b isotype control in combination with rIL-2, rIL-18, or both, twice a week for 4 weeks. To assess the role of T-cell subsets, mice were administered antimouse CD8, antimouse CD4, and antimouse CCL5 Abs (BioXcell). αCD8Ab, αCD4Ab, αCCL5Ab, and αasialoGM1Ab (Wako) were intraperitoneally injected twice a week for 4 weeks, 1 day before. and 1 day after the administration of rIL-2+rIL-18+α-PD-L1Ab.

### CT analysis

To assess changes in liver tumor size, *Mdr2-*KO mice underwent 3D micro-CT imaging with the CosmoScan GX system (Rigaku, Japan). The mice were intravenously injected with 100 µL of the contrast agent ExiTron nano 12000 (Miltenyi Biotec, Germany) 24 h before imaging. Animals were anesthetized and secured in plastic holders during scanning.

### Histological analysis of liver tissue samples

Livers were fixed in 10% formalin, sectioned, and stained with H&E. Immunohistochemistry for PD-L1, CD4, and CD8 was performed using anti-PD-L1 (proteintech Japan, Tokyo, Japan), αCD4 (CAL4, Abcam, Cambridge, UK), and αCD8 (Santa Cruz Biotechnology, Santa Cruz, CA, USA) Abs, followed by detection using the VECTASTAIN Elite ABC Kit (Vector Laboratories). Apoptotic cells were detected using the terminal TUNEL assay (DeadEnd Colorimetric TUNEL System, Promega, Madison, WI, USA). Tissue sections were digitally imaged using a NanoZoomer C9600-03 (Hamamatsu Photonics K.K., Hamamatsu, Japan) for further analysis.

### Immunofluorescence imaging

MDRHC2 cells were analyzed using immunofluorescence staining. Primary Abs included antirabbit AFP (Proteintech, Rosemont, IL, USA) and antigoat albumin (R&D Systems). Secondary Abs included Alexa Fluor 488-conjugated antigoat IgG and Alexa Fluor 555-conjugated antirabbit IgG. Nuclei were stained with DAPI (Thermo Fisher Scientific, Waltham, MA, USA). Imaging was performed using a BZ-X710 fluorescence microscope (Keyence, Japan).

### Measurement of serum cytokine, chemokine, AFP, IL-18BP, and ALT levels

Serum cytokine and chemokine levels were measured using the Bio-Plex mouse Cytokine 31-Plex Panel, Chemokine Panel, and MMP Panel (Bio-Rad Laboratories), according to the manufacturer's instructions. Samples were analyzed in 96-well plates using the Bio-Plex Suspension Array System and Bio-Plex Manager software (Bio-Rad Laboratories). For serum-based analyses, an independent cohort of mice was used (control *n* = 4, treatment *n* = 8), distinct from the cohort used for cellular analyses. Mouse serum AFP levels were measured using the Mouse AFP Quantikine ELISA Kit (R&D Systems, Minneapolis, MN, USA), and IL-18BP levels were assessed using a mouse IL-18BP ELISA Kit (R&D Systems). Serum ALT levels were measured using Fuji Dry Chem Slide ALT-P (Fujifilm, Osaka, Japan).

### Cell isolation

Splenocytes were isolated by pressing spleens through 70 µm cell strainers (BD Biosciences). Intrahepatic leukocytes were isolated from livers perfused with phosphate-buffered saline (PBS) via the inferior vena cava. Perfused livers were digested in 10 mL RPMI 1640 medium (Nissui Pharmaceutical Co., Ltd.) containing 0.02% (w/v) collagenase IV (Sigma-Aldrich) and 0.002% (w/v) DNase I (Sigma-Aldrich) for 40 min at 37 °C. The digested suspensions were layered over lympholyte-M (Cedarlane) and centrifuged at 1,000 × *g* for 20 min. Leukocytes at the interface were collected, washed with PBS, and centrifuged at 500 × *g* for 5 min. Pellets were resuspended in 1 mL of RPMI 1640 containing 10% fetal bovine serum (FBS).

### Flow cytometry analysis

Intrahepatic and splenic leukocytes were cultured in 96-well round-bottom plates (200 µL/well) at 37 °C for 4 h in RPMI 1640 supplemented with human recombinant IL-2 (50 U) and 0.2 mL of BD GolgiPlug protein transport inhibitor (BD Biosciences). For IFN-γ production assays, cells were stimulated with PMA (20 ng/mL; Sigma) and ionomycin (1 µg/mL; Sigma) in the presence of GolgiPlug. Following stimulation, cells were harvested, washed in PBS containing 1% FBS, incubated for 10 min on ice, blocked with unlabeled antimouse CD16/32 Ab (BD Biosciences; to block binding to Fc RII/III), and stained with surface Abs for 20 min on ice. Abs included allophycocyanin (APC)-conjugated anti-CD8, CD4, and NK1.1; fluorescein isothiocyanate (FITC)-conjugated monoclonal antimouse CD69, PD-1, and CD25 Abs; and phycoerythrin (PE)-conjugated Abs against Foxp3 and IFN-γ (all from BD Biosciences). Data were acquired using a FACSCalibur (BD Immunocytometry Systems, San Jose, CA, USA) and analyzed them using CellQuest and FlowJo v. 10 software (Tree Star, San Carlos, CA, USA).

### RT-qPCR

Total RNA was extracted from liver tissue samples or cultured cells using the RNeasy Mini Kit (Qiagen, Valencia, CA, USA), followed by DNase treatment. Reverse transcription was performed using the High-Capacity cDNA Reverse Transcription Kit (Applied Biosystems, Foster City, CA, USA). Quantitative PCR was conducted using TaqPath qPCR Master Mix (Applied Biosystems) and CG formulation (Thermo Fisher Scientific) on a LightCycler 480 system (Roche Applied Science, Mannheim, Germany). Gene expression was analyzed using TaqMan probes for *Ccl5* (Mm01302427_m1) and *Gapdh* (Mm99999915_g1). Expression levels were normalized to GAPDH levels in each sample.

### Xenium in situ gene expression analysis

In situ transcriptomics was performed using the Xenium platform (10× Genomics) according to the manufacturer's protocols. Five-micrometer-thick sections were prepared from formalin-fixed paraffin-embedded (FFPE) tissue blocks following the *Xenium In Situ for FFPE-Tissue Preparation Guide* (CG000578 Rev C). Sections were mounted on Xenium slides (PN-1000465), deparaffinized, and permeabilized as per the *Fixation and Permeabilization Protocol* (CG000580 Rev C).

Probe hybridization was performed using the 10× Genomics Mouse Multi-Tissue Atlassing Panel (379 predesigned genes) supplemented with a custom panel containing 100 additional target genes, yielding a total of 479 RNA targets (Design ID: XC7BTF), created using the Xenium Panel Designer per the *Add-on Panel Design Technical Note* (CG000643 Rev B). Cell segmentation was performed using Xenium *In Situ* Cell Segmentation Reagents (PN-100661), which include multi-tissue stains for membrane, cytoplasm, ribosomal RNA, and nuclear [4',6-diamidino-2-phenylindole (DAPI)] labeling.

Following hybridization, ligation, amplification, staining, and autofluorescence quenching, slides were imaged using the Xenium Analyzer, per the *Decoding and Imaging User Guide* (CG000584 Rev E). Analyzer version 3.0.2.0 and Analysis Software version 3.0.0.15 were used in this study. The resulting data were analyzed using Xenium Explorer version 3.1.0. H&E staining was subsequently performed on the Xenium slides. Quality control was confirmed using the Xenium Analyzer's output summary HTML report, which showed no errors.

We performed downstream data analysis in R (version 4.2.2) using the Seurat (version 4.3.0), Monocle3 (version 1.2.9), and tradeSeq (version 1.14.0) packages. Cell-by-gene count matrices from the Xenium platform were imported into R and processed using SCTransform normalization. This was followed by principal component analysis, UMAP (dims = 1:30), and Leiden clustering (resolution = 0.6). These steps initially identified 18 distinct clusters. The clusters were annotated based on canonical marker genes and consolidated into 13 transcriptionally defined cell types: hepatocytes, liver tumor cells, endothelial cells, hepatic stellate cells, Kupffer cells, cholangiocytes, monocytes, fibroblasts, B cells, neutrophils, proliferating cells, pericytes, and T cells.

To focus on T cells, we extracted 21,857 cells expressing ≥3 UMIs of Cd3d, Cd4, or Cd8a from the full dataset. We then renormalized and reclustered these cells at a resolution of 0.8, yielding 13 subclusters. These were further grouped into six canonical functional subsets: CD4^+^ naive/Tcm, CD4^+^ Tcm, CD4^+^ Treg, CD8^+^ Tcm, CD8^+^ Tem, and CD8^+^ Tex. Pseudotime trajectory analysis was performed using Monocle3, with the root node assigned based on CD8^+^ Tcm. TradeSeq was applied to identify genes showing condition-specific expression dynamics along pseudotime; Ccl5 was identified as a top hit. No custom code was used beyond package defaults.

### Statistical analysis

Data are expressed as mean ± SD for the data collected from each experiment. The means of two groups were compared using a two-tailed Student's t test, and the means of multiple groups were compared using one-way ANOVA followed by Bonferroni's post hoc tests using GraphPad Prism 10.0 (GraphPad Prism Inc., San Diego, CA, USA). Statistical significance was set at *P* < 0.05.

## Supplementary Material

pgag113_Supplementary_Data

## Data Availability

The spatial transcriptomic data generated in this study have been deposited in the DDBJ Genomic Expression Archive (GEA) under experiment accession number E-GEAD-1176. The data will be publicly available at the time of publication and are currently accessible via the DDBJ GEA reviewer access system. All other data are available within the manuscript and its Supplementary information. All analyses were conducted using standard packages (Seurat, Monocle3, and tradeSeq), and no custom code was used.
