# Supplementary material for: Combined administration of interleukin-2 and 18 with anti-PD-L1 antibody induces CCL5-positive CD8 T cells to suppress liver tumors
Source: PNAS Nexus. 2026 Apr 15;5(4):pgag113. doi: 10.1093/pnasnexus/pgag113 (PMC13096734; doi:10.1093/pnasnexus/pgag113)
Supplement: pgag113_Supplementary_Data [file pgag113_supplementary_data.docx]

Supplementary Materials for

**Combined administration of interleukin-2 and 18 with anti-PD-L1 antibody induces CCL5-positive CD8 T cells to suppress liver tumors**

Masamichi Kimura^1^, Kenzaburo Yamaji^2^, Kenichi Harada^3^, Hideya Kawaji^4^, Jun Imamura^1^, Haruki Okamura^5^, Yoshimasa Tanaka^6^, Michinori Kohara^2^,　Kiminori Kimura^1*^

* Corresponding author: [kiminori_kimura@tmhp.jp](mailto:kiminori_kimura@tmhp.jp)

**This PDF file includes**

Supplemental Fig. 1 to Fig. 5

**SI Figure 1. rIL-2+rIL-18+**α**PD-L1Ab induces inflammation via cytokine and chemokine production.**

**(A-B)** Serum ALT, cytokines and chemokines were measured using specimens from each group 4 weeks after treatment. Data represent pooled biological replicates from the tumor assessment cohort and additional independent experiments. Total sample sizes: control (n = 14), rIL-2+αPD-L1Ab (n = 6), rIL-18+αPD-L1Ab (n = 12), triple therapy (n = 18). Data are presented as mean ± SD. * *p* < 0.05; ***p* < 0.01; ****p* < 0.005, using one-way ANOVA followed by Bonferroni’s post-hoc test.

**SI Figure 2. rIL-2+rIL-18 alone does not induce significant antitumor effects on *Mdr2*-KO mice.** **(A)** Male *Mdr2-*KO mice aged 78–96 weeks were used in the control and rIL-2+rIL-18 or rIL-2+rIL-18+αPD-L1Ab treatment groups. **(B)** Forty-eight hours before the first administration, *Mdr2-*KO mice were injected with a contrast agent (100 μL/mouse) and CT was performed 24 h later to determine the tumor size and location in the liver. Histological examination of liver tissues using H&E staining after treatment (scale bars, 100 μm). **(C)** Comparison of tumor sizes before and after administration, with the largest tumor discernible on imaging. **(D, E)** The production capacity of CD8^+^ T cell-derived IFN-γ in the rIL-2+rIL-18+αPD-1Ab-treated group was compared with that in the control group. IHLs were stimulated using PMA+ionomycin, and the IFN-γ production ability of CD8^+^ cells was analyzed. **(F)** mRNA expression of *Ccl5* in the liver as determined by RT-qPCR. Data are presented as mean ± SD. * *p* < 0.05; ***p* < 0.01; ****p* < 0.005; *****p* < 0.001; n.s. not significant vs. control-treated *Mdr2-*KO mice, using one-way ANOVA followed by Bonferroni’s post-hoc test.

**SI Figure 3.** **Spatial expression of Cd8a, Ifng, and Il18r1 in control and treated livers.** (A) Representative Xenium spatial transcriptomic images showing Cd8a, Ifng, and Il18r1 expression in tumor, border zone, and non-tumor regions from control and triple therapy–treated mice. (B) Violin plots (with internal box-and-whisker overlays) showing single-cell Ifng and Il18r1 expression (log₁p-normalized counts) in CD8⁺ Tem cells isolated from four Xenium ROIs. Horizontal brackets indicate within-region comparisons (two-sided Wilcoxon rank-sum test with Bonferroni correction); **** p < 0.001.

**SI Figure 4. Antitumor effect of rIL-2+rIL-18+αPD-L1Ab administered for 8 weeks.** (**A**) Male *Mdr-2*KO mice (78–96 weeks old) were intraperitoneally administered rat IgG2b (as control) or rIL-2+rIL-18+αPD-L1Ab (n = 5 per group) for 8 weeks. (**B**) Forty-eight hours before the first administration, *Mdr2-*KO mice were injected with a contrast agent (100 μL/mouse); 24 h later, CT was performed to determine the tumor size and location in the liver. (**C, D**) Comparison of tumor size before and after administration, with the largest tumor discernible on imaging. **(E, F)** The production capacity of CD8^+^ T cell-derived IFN-γ in the rIL-2+rIL-18+αPD-L1Ab-treated group was compared with that in the control group. IHLs were stimulated with PMA and ionomycin, and the IFN-γ production ability of CD8+ T cells was analyzed. Data are presented as mean ± SD. **p* < 0.05; ***p* < 0.01; ****p* < 0.005; n.s. not significant vs. control-treated *Mdr2-*KO mice, as determined using Student’s *t*-test.

**SI Figure 5.** **Comparable antitumor effects of rIL-2+rIL-18 combined with αPD-1Ab in Mdr2-KO mice.** Male *Mdr2-*KO mice aged 78–96 weeks were used in both the control and rIL-2+rIL-18+αPD-1Ab treatment groups. **(A)** Schematic of treatment protocol. **(B)** Forty-eight hours before the first administration, *Mdr2-*KO mice were injected with a contrast agent (100 μL/mouse), and CT was performed 24 h later to determine tumor size and location in the liver. Liver histology using H&E staining after treatment administration (scale bars, 100 μm). **(C) Comparison of** tumor size before and after administration,　with the largest tumor discernible on imaging. **(D, E)** The production capacity of CD8^+^ T cell-derived IFN-γ in the rIL-2+rIL-18+αPD-1Ab-treated group was compared with that in the control group. IHLs were stimulated using PMA and ionomycin, and the IFN-γ production ability of CD8^+^ T cells was analyzed. Data are presented as mean ± SD. **p* < 0.05; ***p* < 0.01; ****p* < 0.005; *****p* < 0.001; n.s. not significant vs. control-treated *Mdr2-*KO mice, as determined using Student’s *t*-test.
